# Comparing the Effects of Chemical Optical Clearing Agents and Mechanical Compression on the Optical Attenuation of Porcine Skin

**DOI:** 10.1002/jbio.70323

**Published:** 2026-07-24

**Authors:** Camila Ramos Silva, Tania Mateus Yoshimura, Jailda Nonato dos Santos Oliveira, Marcus Paulo Raele, Marcos Antonio Hortellani, Martha Simões Ribeiro, Denise Maria Zezell, Marcello Magri Amaral

**Affiliations:** ^1^ Nuclear and Energy Research Institute‐ IPEN/CNEN São Paulo Brazil; ^2^ Biomedical Engineering Universidade Brasil São Paulo Brazil; ^3^ Lumo Life Science Institute for Science, Technology and Innovation São Paulo Brazil

**Keywords:** fructose, light attenuation, polyethylene glycol, skin clearing, tartrazine

## Abstract

Light scattering remains a primary challenge for deep‐tissue optical diagnostics and therapies. While Optical Clearing Agents (OCAs) are used to reduce skin attenuation, Mechanical Compression (MC) is also applied in clinical settings despite its inconclusive results. Their relative effectiveness under standardized conditions remains poorly characterized. This study quantitatively compares various OCA formulations, including mineral oil, PEG‐400, fructose, and tartrazine, with enhancers such as oleic acid and 1,2‐propanediol, under load‐dependent MC (1.6–47 kPa). Using spectral‐domain OCT at 840 nm on porcine skin, we found that PEG‐400/oleic acid (80/20%) achieved the highest efficiency, reducing the optical attenuation coefficient (OAC) by 23.8% ± 1.7% within 15 min. Fructose‐based and aqueous tartrazine formulations also showed significant reductions (16.8% and 20.4%). Conversely, our results show that MC does not effectively reduce skin optical attenuation. These findings demonstrate that OCAs provide a more potent, stable reduction in OAC, offering a superior strategy for optimizing light delivery.

## Graphical abstract

Quantitative SD‐OCT analysis reveals that topically applied chemical optical clearing agents, specifically PEG‐400 combined with oleic acid and aqueous tartrazine, significantly reduce the skin optical attenuation coefficient. In contrast, mechanical compression proved ineffective, identifying chemical clearing as a superior strategy for optimizing light delivery through the skin in clinical biophotonics.

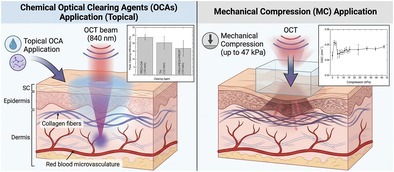

## 1 Introduction

Overcoming the intrinsic scattering limits of biological tissues remains a cornerstone for advancing deep‐tissue optical diagnostics and laser‐based therapies. The light‐tissue interaction depends on the wavelength and intensity of the incident radiation, as well as the spatial distribution of endogenous chromophores. In the optical range, this interaction is primarily governed by absorption and scattering, which are characterized by the absorption (*μ*_a_) and the scattering (*μ*_s_) coefficients, respectively. These parameters ultimately limit the depth of light penetration and the performance of diagnostic (imaging or spectroscopic) and therapeutic techniques [1, 2].

Absorption arises from endogenous chromophores such as hemoglobin, melanin, water, and lipids, each of which exhibits wavelength‐dependent spectral features [2, 3]. For instance, hemoglobin strongly absorbs light below 600 nm, while melanin dominates the ultraviolet and early visible regions of the spectrum. In the near‐infrared, water absorption increases markedly above 1350 nm, and lipids show prominent absorption bands between 1200 and 1700 nm [4].

Meanwhile, scattering results from refractive‐index mismatching between subcellular structures and the surrounding interstitial medium [5, 6, 7]. This mismatching occurs across distinct components, such as cell membranes and organelles (e.g., mitochondria and lysosomes), which present a high density of lipid structures [5, 6, 8], scleroprotein fibrous bundles, predominantly type I collagen and elastin fibers in the dermis layer [6, 9, 10], and the surrounding medium, where the baseline mismatch in determined by the refractive index of the extracellular or interstitial fluid [6, 7, 11]. A greater refractive index mismatch leads to higher scattering and, consequently, increased light attenuation. Scattering intensity is also wavelength‐dependent, decreasing with the wavelength (μ_s_ ∝ λ). Therefore, shorter wavelengths, such as blue or green light, experience significantly more scattering than longer wavelengths in the red or near‐infrared (NIR) range [1].

The interplay between the spectral dependence of scattering and absorption gives rise to the optical therapeutic window (650–1350 nm), a wavelength range where both phenomena are relatively minimized. Within this window, optical attenuation is primarily governed by scattering rather than absorption. This reduced attenuation allows light to penetrate deeper into biological tissues with minimal interference from endogenous chromophores, enabling the effective use of technologies such as Optical Coherence Tomography (OCT) [6, 8, 12, 13, 14, 15, 16, 17], spectroscopy [6, 18], photoacoustic microscopy [19, 20, 21, 22, 23], as well as therapeutic modalities like photobiomodulation (PBM) and photodynamic therapies (PDT) [7, 24].

In therapeutic applications, residual attenuation imposes a critical constraint on the irradiance delivered to deep‐seated target tissues. This limitation often necessitates higher incident powers to achieve the desired biological effect at depth, which may increase the risk of thermal damage to superficial layers. Similarly, in imaging techniques such as OCT, high scattering coefficients significantly restrict the maximum depth at which internal structures can be clearly visualized, often limiting the imaging range to the superficial layers of the skin or mucosa.

To minimize light scattering and increase penetration depth, several strategies have been explored. In clinical practice, a widely adopted approach in optical therapeutic techniques is the application of mechanical compression (MC) on the skin surface [25]. This method is intended to reduce the thickness of superficial tissue layers. It is often proposed that the MC promotes the outflow of interstitial fluid and displaces blood from the microvasculature. Consequently, light delivery to deeper tissues may be facilitated by a shorter optical path length, improved refractive‐index matching, and reduced hemoglobin‐dependent absorption and scattering [5, 26, 27].

However, the efficacy of this approach remains a matter of debate within the scientific community [5, 27]. These effects are often transient and may induce localized tissue trauma and structural distortions, potentially compromising tissue integrity and morphological fidelity required for an accurate imaging analysis.

Another prominent approach for reducing attenuation is the use of Optical Clearing Agents (OCAs). OCAs are substances specifically selected to reduce scattering in biological tissues. By decreasing scattering, these agents facilitate deeper light penetration, enhancing depth resolution and contrast in optical imaging methods such as OCT [8, 13, 14, 15, 16, 17], confocal microscopy [28], and two‐photon microscopy [29], which are typically limited by the tissue optical properties. Additionally, this approach can significantly increase light exposure at various depths during light‐based treatments [17].

Generally, the three primary mechanisms for OCA's action are refractive index‐matching, tissue dehydration, and reversible dissociation of collagen fibers [6]. The most common OCAs for skin applications, particularly in vivo, include glycerol, glucose, fructose, and polyethylene glycol (PEG), chosen for their recognized biocompatibility and favorable pharmacokinetics [6]. However, the effectiveness of an OCA is not determined solely by its refractive index or osmolarity [30]: its capacity for tissue permeation is equally critical [7, 18, 31]. Consequently, to achieve effective penetration at biocompatible concentrations, OCAs are often combined with chemical permeation enhancers, such as propanediol (PROP), dimethyl sulfoxide (DMSO), oleic acid (OA), ethanol, azones, thiazone [10, 18, 20], or assisted by physical methods including ultrasound, microneedles, and tissue compression [32, 33, 34]. Aqueous tartrazine (TA) was recently identified as a potent OCA by Ou et al. [35]. By absorbing blue light, TA elevates the refractive index at longer wavelengths via Kramers–Kronig relations, enhancing index matching with tissue components. Nevertheless, because previous investigations utilized intradermal injections, the effectiveness of TA as a topical clearing agent has not yet been established.

Despite the relevance of both MC and chemical OCAs, no study has directly compared their performance in reducing light attenuation using similar experimental conditions. Therefore, this study aims to evaluate and compare the effectiveness of either various OCA formulations or mechanical pressure levels on the optical attenuation coefficient (OAC) of porcine skin. By employing OCT, we sought to determine which of these two distinct approaches provides a more robust reduction in attenuation, thereby identifying the most efficient strategy for enhancing diagnostic and therapeutic light delivery.

## 2 Materials & Methods

### 2.1 Reagents

The chemical optical clearing agents used in this study included OA (analytical grade, CAS No. 112‐80‐1, Cat. No. R01661000, ACS Científica, Brazil); PEG‐400k (analytical grade, CAS No. 25322‐68‐3, Cat. No. PG07365RA, Êxodo Científica, Brazil); mineral oil (MO, analytical grade, CAS No. 8042‐47‐5, Infinity Pharma, Brazil); D‐fructose (FRU, analytical grade, powder, CAS No. 57‐48‐7, Cat. No. R07971000, ACS Científica, Brazil); 1,2‐propanediol (PROP, 96% purity, CAS No. 4254‐14‐2, Cat. No. 540242, Sigma‐Aldrich, Germany); and TA (≥ 85% purity, powder, CAS No. 1934‐21‐0, Cat. No. T0388, Sigma‐Aldrich, USA).

### 2.2 OCA Formulations

The OCA solutions and formulations were prepared at room temperature, unless otherwise stated and under constant magnetic stirring.

Aqueous FRU solution was prepared at 78% (w/v) in distilled water under mild heating (40°C), while TA was vigorously vortex‐mixed in distilled water (30 w/w%), heated at 80°C until complete dissolution, and then stored at 37°C to ensure it remained in a liquid state.

The experimental groups comprised single‐agent OCAs, including neat OA, PEG, and MO, as well as the TA solution. Additionally, four OCA blends were evaluated at concentrations previously shown to enhance light penetration in skin during OCT imaging [13, 17, 36]. These formulations included PEG+OA, PEG+PROP, FRU+PROP, and FRU+PEG+PROP, and their concentration ratios are provided in Table 1.

**TABLE 1:** Optical clearing agents (OCA) formulation and concentration ratios.

Formulation	Concentration ratio, % (v/v)
Oleic Acid (OA)	100
Polyethylene Glycol 400k (PEG)	100
Mineral Oil (MO)	100
Tartrazine (TA)	100
PEG+OA	80/20
PEG+PROP	95/5
FRU+PROP	95/5
FRU+PEG+PROP	55/40/5

### 2.3 Experimental Model

Porcine skin was selected as the experimental model due to its well‐documented histological and physiological similarities to human skin, particularly regarding epidermal thickness, dermal collagen arrangement, and vascularization patterns [37, 38]. As an accessible and cost‐effective surrogate, it serves as a gold standard for ex vivo optical and permeation studies, effectively mimicking human skin conditions in fields such as dermatology and tissue engineering.

To minimize the intrinsic variability of biological tissue, a single large specimen of fresh porcine belly (ventral abdomen) skin was sourced from a local commercial supplier to ensure uniformity across replicates. The porcine skin specimens were stored frozen until use. Before the experiments, samples were thawed at 4°C and maintained at 22°C ± 4°C with 60% ± 5% relative humidity during the experiment to ensure standardized environmental conditions. The bulk tissue was partitioned into 5 × 5 cm sections and maintained under refrigeration until use. Following experimental procedures, samples were discarded according to sanitary regulations.

### 2.4 OCT System and Data Acquisition

Imaging acquisition was performed using a Spectral Domain OCT (SD‐OCT) system (OQ LabScope XR 2.0, Lumedica, USA) operating at a central wavelength of 840 nm. The system provides an axial and lateral resolution of approximately 5 μm and 15 μm, respectively. Data were captured at an A‐scan rate of 34 kHz. For each experimental condition, 30 B‐scan images (512 × 512 pixels) were acquired and subsequently averaged to minimize noise and optimize the signal‐to‐noise ratio.

All measurements were performed in triplicate to ensure reproducibility. From the average image, the OAC was estimated using a custom‐developed software at two distinct positions within each B‐scan using MATLAB. Briefly, in every B‐scan, two regions of 10 A‐scans were averaged to reduce the noise effect after alignment through the skin surface. The exponential decay model ($I\left(z\right)=A\exp\left(-2\mathrm{\mu z}\right)$) was applied to the averaged A‐scan to estimate the OAC (μ). Furthermore, all images were captured at a fixed focal distance to maintain signal consistency across the different experimental groups.

### 2.5 OCA Administration and Mechanical Compression Protocols

Baseline OCT images were acquired for each skin specimen prior to the application of any OCA or mechanical compression. These pre‐treatment images served as internal controls.

The OCA's formulations were topically applied to the epidermal surface of the skin sections in a standardized volume of 1000 μL and were manually rubbed for 3 min. This point was defined as experimental time 0 min. After removing excess agent from the skin surface with a tissue wipe, images were captured every 3 min for 15 min, totaling 6 time points (0, 3, 6, 9, 12, and 15 min), in addition to the internal control group. The 15 min experimental window was selected based on the clinical relevance of a rapid‐clearing effective protocol.

**FIGURE 1:**
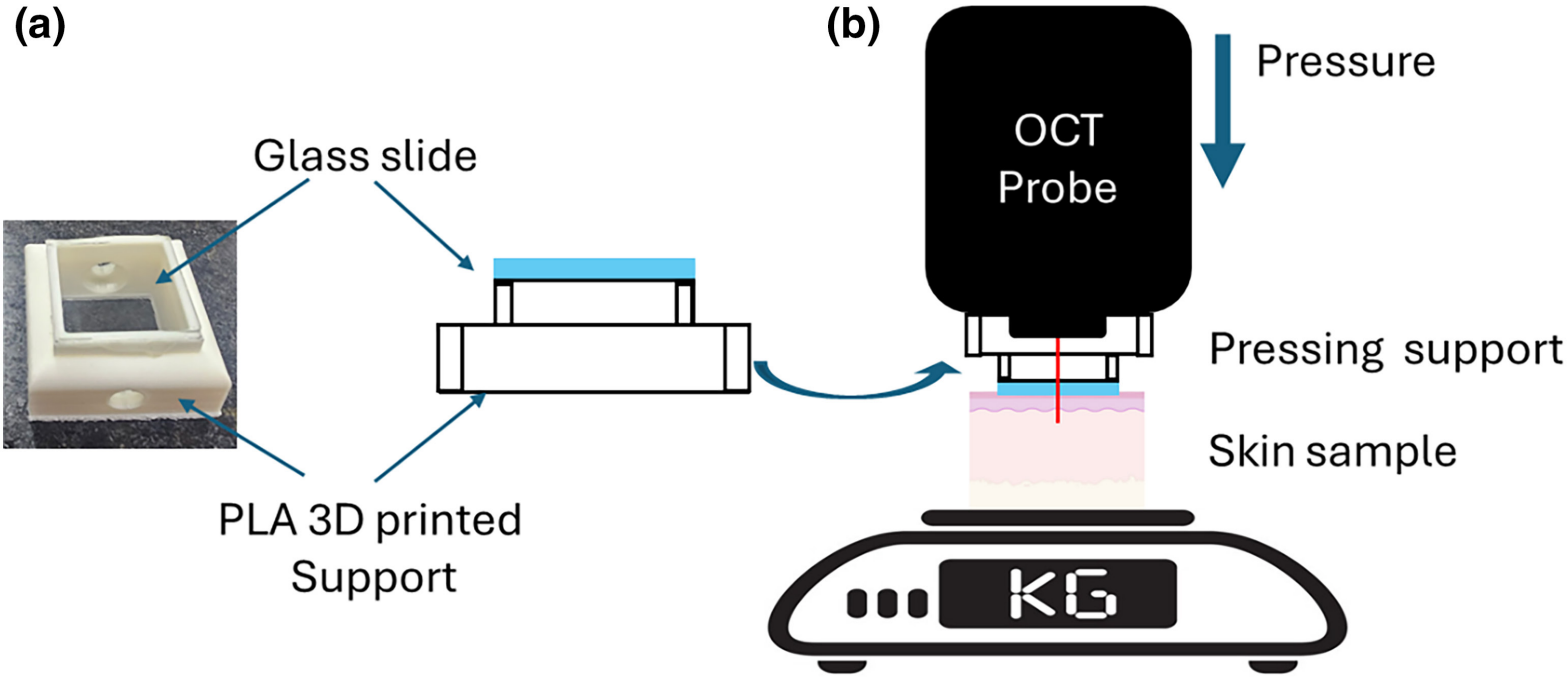
Custom‐made OCT support for controlled mechanical compression application. (a) Detail of the 3D‐printed PLA support featuring a 25 × 25 mm glass window for simultaneous compression and imaging acquisition. (b) Schematic representation of the experimental setup. The OCT probe is coupled to the pressing support, which exerts a downward force on the skin sample. The compression is monitored via a calibrated digital scale.

To apply uniform pressure to the skin during simultaneous OCT image acquisition, a custom‐made support system was developed and 3D printed using polylactic acid (PLA) (Figure 1a). A microscope slide was cut and UV‐glued onto the support to create a 25 × 25 mm optical window, ensuring constant pressure distribution across the region of interest. To avoid reflection artifacts at the glass–tissue interface, the image processing included the removal of the initial saturated pixels, ensuring analysis within pure skin tissue. The skin samples were placed on a calibrated scale to monitor the applied force, from which the corresponding pressure was calculated (Figure 1b). OCT images were acquired under decreasing compression levels, starting at 3 kg (~47 kPa) and reducing in 0.5 kg (~7.8 kPa) increments down to 1 kg (~15.7 kPa). For values below 1 kg, the compression was reduced in 0.1 kg intervals until reaching 0.1 kg (~1.6 kPa). Once compression was applied, data acquisition was delayed until the pressure stabilized to guarantee that tissue accommodation and elasticity did not influence the measurements.

### 2.6 Statistical Analysis

The distribution of the data for each assay was assessed using the Shapiro–Wilk test in OriginPro 2018. Group comparisons were performed using one‐way repeated‐measures ANOVA, followed by Fisher's Least Significant Difference (LSD) post hoc test. All experiments were conducted in triplicate on three separate days. For each image, two distinct regions were selected to calculate the optical attenuation coefficient, yielding a *n* = 6. Statistical significance was defined as *p* < 0.05.

## 3 Results

### 3.1 Control Group Over Time

**FIGURE 2:**
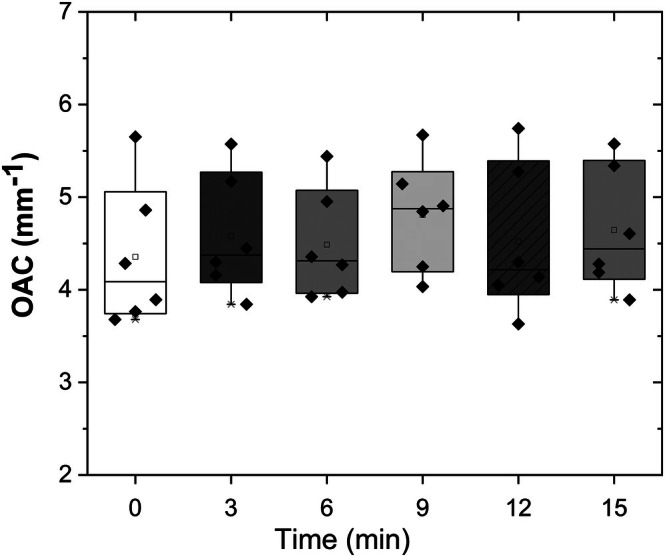
Temporal stability of the optical attenuation coefficient (OAC) for the control group over a 15 min period. Boxplots display the median (line), mean (square), interquartile range (box), and minimum and maximum (whiskers). No statistically significant differences were observed among time points.

The OAC of the control group (no treatment) was monitored for 15 min to verify its time stability (Figure 2). As expected, in the absence of external intervention, the OAC remained constant across all experimental time points, maintaining the median approximately at 4.5 mm^−1^. Given this stability, the pre‐treatment baseline was validated as a reference for quantifying treatment‐induced optical changes.

### 3.2 Effects of Individual and Combined Optical Clearing Agents

**FIGURE 3:**
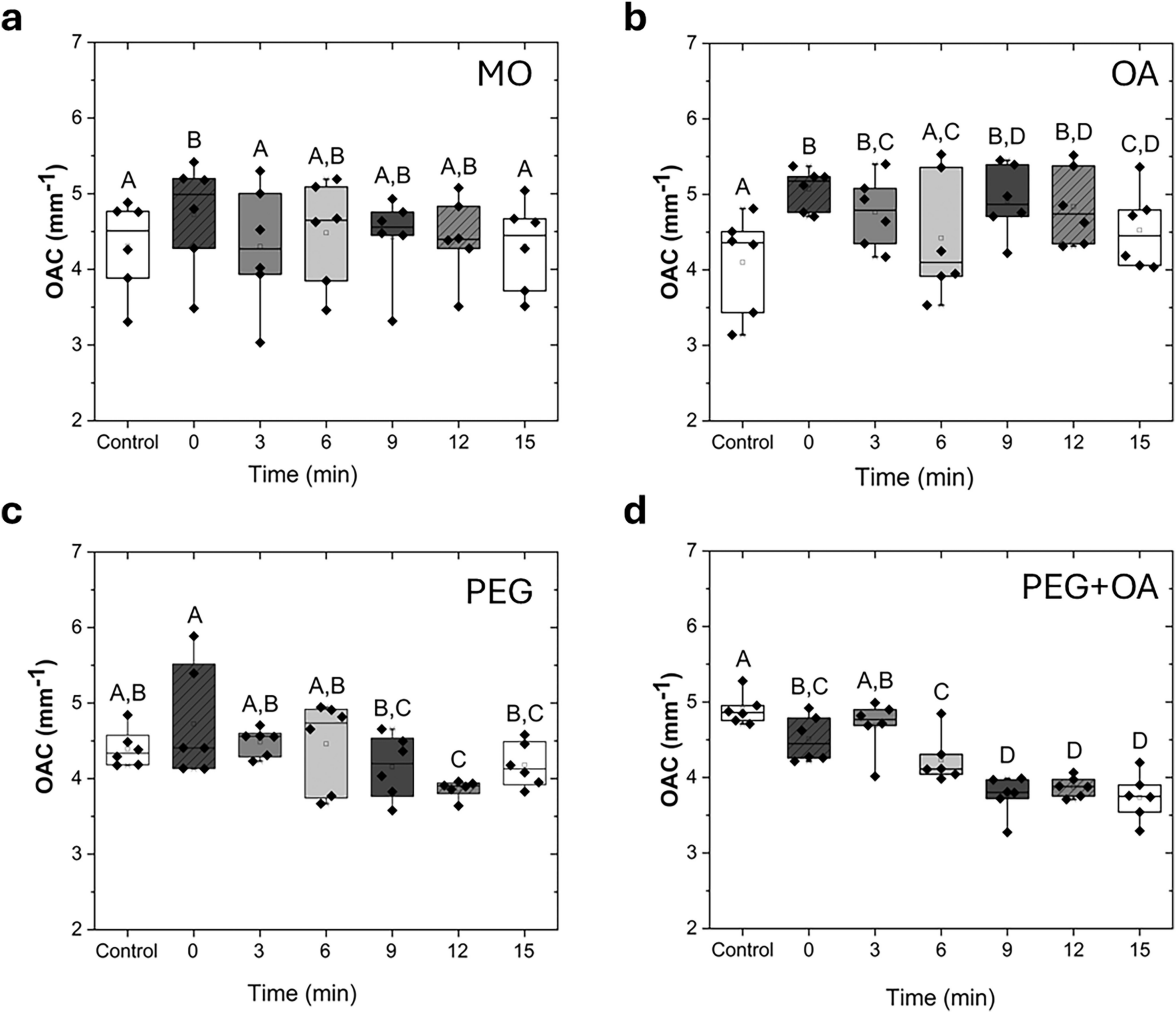
Temporal variation of optical attenuation coefficient (OAC) for different OCA treatments. (a) Mineral Oil (MO); (b) Oleic Acid (OA); (c) Polyethylene Glycol 400k (PEG), and (d) PEG+OA. Boxplots display the median (line), mean (square), interquartile range (box), and minimum and maximum (whiskers). Different capital letters indicate statistically significant differences within each experimental group (*p* < 0.05).

The application of different clearing agents revealed distinct optical attenuation profiles depending on the substance used. MO induced a statistically significant 10.3% increase in OAC at 0‐min compared to the control group, subsequently returning to its baseline value (Figure 3a).

For OA, nearly all experimental datapoints showed an increase in OAC relative to the control group, ranging from 12.0% to 25.8% (at 15 and 0 min, respectively), except for the 6 min interval (Figure 3b). Significant internal variation was also detected. The OAC at 0 min was higher than both 6 and 15 min, while the 6 min point recorded a lower mean value than those at 9 and 12 min.

In contrast, PEG induced an 11.8% decrease in OAC at 12 min compared to the control group (Figure 3c). This time point represents the most pronounced effect for this agent and was also significantly different from the 0, 3, and 6 min intervals. Notably, the 0 min interval exhibited higher optical attenuation than the 9, 12, and 15 min points.

When PEG was combined with a permeation enhancer (PEG+OA), a significant decrease in OAC was observed at 0 min and then at 6 min relative to the control group (Figure 3d). This trend of reduced attenuation persisted throughout the remainder of the experiment, with the 9, 12, and 15 min intervals showing statistically similar responses, with the largest reduction observed at 15 min (23.8%).

**FIGURE 4:**
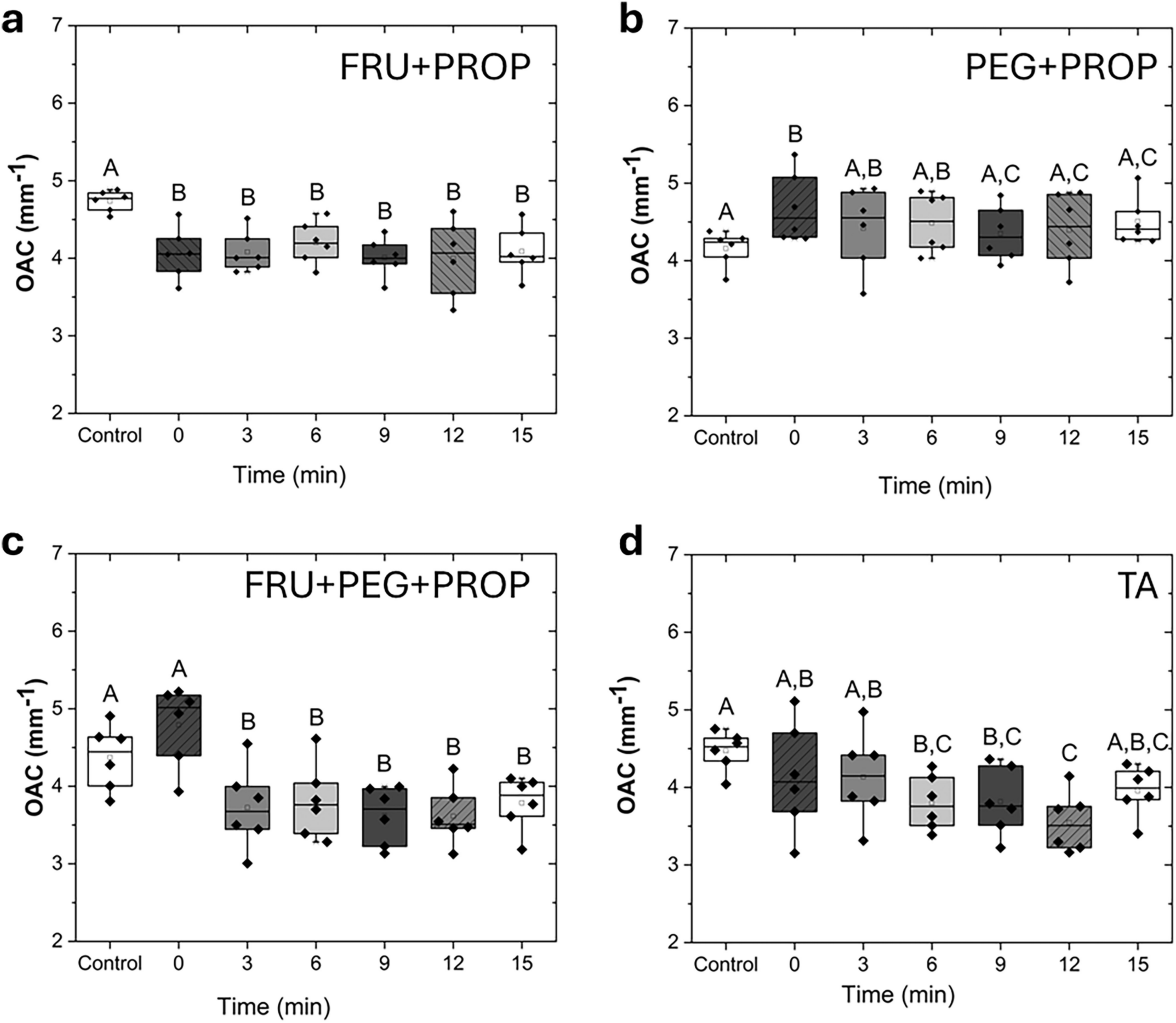
Temporal variation of the optical attenuation coefficient for complex OCA formulation and tartrazine. (a) fructose and propanediol (FRU+PROP), (b) polyethylene glycol 400k and propanediol (PEG+PROP), (c) fructose, polyethylene glycol 400k and propanediol (FRU+PEG+PROP), and (d) Tartrazine (TA). Boxplots display the median (line), mean (square), interquartile range (box), and minimum and maximum (whiskers). Different capital letters indicate statistically significant differences within each experimental group (*p* < 0.05).

The FRU+PROP combination exhibited a statistically significant reduction in the OAC (ranging from 11.2% to 15.7%) compared to the control group throughout the entire experimental period (Figure 4a). Notably, no significant variation was observed among the internal time points within this group, indicating a stable clearing effect.

In contrast, the PEG+PROP combination showed a significantly higher OAC than the control group only at the initial 0 min time point (13.4%; Figure 4b). At subsequent intervals, this difference was not sustained as OAC values became comparable to those of the control group. These findings suggest a transient increase in light attenuation followed by a stabilization at baseline levels.

Conversely, the addition of fructose to the PEG+PROP mixture (FRU+PEG+PROP) maintained a sustained reduction in the OAC over time, with a statistically significant difference relative to the control group from 3 min onward, with the maximum reduction (16.8%) observed at 12 min (Figure 4c).

Regarding the TA group, the first two time points did not significantly affect the OAC relative to the control (Figure 4d). However, a statistically significant reduction was exhibited during the 6 to 12‐min interval. When comparing internal experimental times, the 12‐min mark showed the most pronounced reduction (20.4%) and differed significantly from the 0‐ and 3‐min points. By 15 min, a recovery was observed, with no significant difference found compared to any experimental time point or the control condition. Although tartrazine exhibits a strong absorption band in the visible (~428 nm), its effectiveness as a clearing agent in the OCT 840 nm band is mainly due to changes in the refractive index of the interstitial fluid. Strong absorption in the visible influences the refractive index at longer wavelengths (Kramers–Kronig relations), promoting more efficient refractive index matching with collagen fibers (*n*≈1.43–1.45), which explains the observed reduction in scattering without interference from significant absorption losses in the near‐infrared.

**FIGURE 5:**
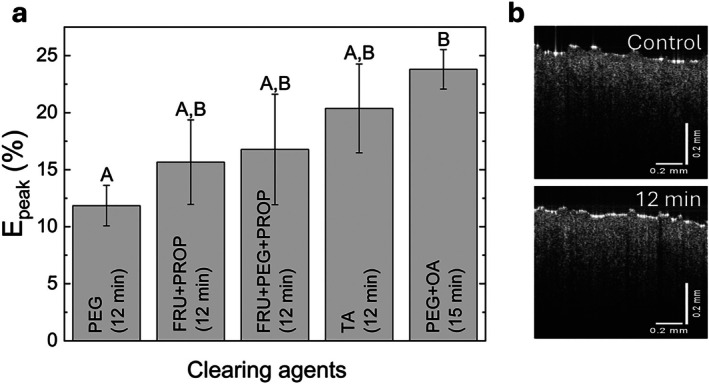
(a) Peak clearing efficiency (E_peak_) comparison across different OCA formulations. The bars represent the maximum percentage reduction in OAC achieved by each agent relative to its respective pre‐treatment baseline. The timepoints indicated in parentheses represent the specific moment within the 15‐min experimental window when the minimum OAC value was attained for that agent. Values are presented as mean ± SEM. Different capital letters indicate statistically significant differences within each experimental group (*p* < 0.05). (b) OCT image of porcine skin before and after 15 min of application of PEG+OA.

To evaluate the relative performance of the different treatments, the peak clearing efficiency (*E*_peak_) was determined by identifying the maximum OAC reduction achieved for each formulation throughout the 15‐min experimental window (Figure 5a). This value was normalized to the respective internal baseline of each specimen to account for initial tissue variability, according to the following equation:

$$E_{\text{peak}}\;\left(\%\right)=\left(\frac{{\mathrm{OAC}}_{\text{baseline}}-{\mathrm{OAC}}_{\min}}{{\mathrm{OAC}}_{\text{baseline}}}\right)\times 100$$

where OAC_baseline_ is the OAC of the native tissue acquired during pre‐treatment scan, and OAC_min_ represents the lowest OAC value recorded for a specific OCA during the 15‐min treatment period. Figure 5b shows an OCT image of the PEG+OA before and after 15 min of application.

### 3.3 Influence of Mechanical Compression (MC) on the OAC

**FIGURE 6:**
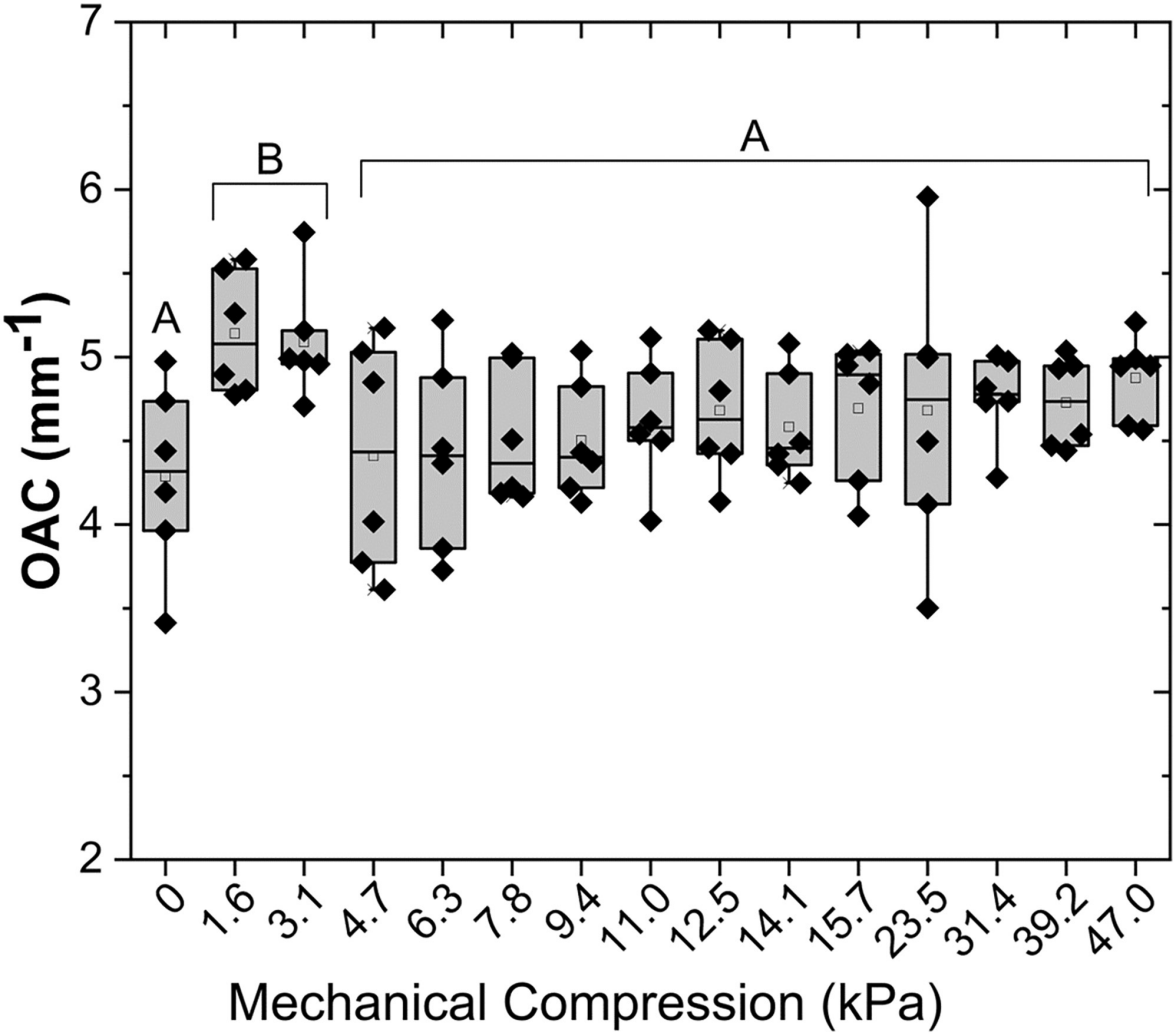
Optical attenuation coefficient (OAC) under varying mechanical compression levels. Boxplots display the median (line), mean (square), interquartile range (box), and minimum and maximum (whiskers). Capital letters indicate statistically significant differences (*p* < 0.05) between load‐compression conditions and the control group (no compression).

A complex, non‐monotonic pattern was observed in the tissue response to mechanical compression. The lowest pressures tested, 1.6 and 3.1 kPa, significantly increased the OAC, whereas intermediate pressures ranging from 4.7 to 23.5 kPa produced no detectable difference compared to the baseline (0 kPa) (Figure 6a). At higher pressure levels, specifically 31.4 and 47 kPa, a trend towards an increase in the median is observed, but it is not statistically significant.

## 4 Discussion

In our experiments, the MC and OCAs were applied topically to the skin surface, mimicking a potential clinical application. Therefore, evaluating the effects of time and permeation dynamics is an important aspect of our study. A 15 min evaluation window was selected to reflect practical clinical scenarios in which rapid action is essential for procedural efficiency and patient comfort. Identifying agents that achieve high peak‐clearing efficiency within this short time frame is important for the development of real‐time optical clearing protocols. Because ex vivo samples were used, we first investigated whether environmental conditions affected the OAC of the control group during the experimental period. The results showed that the OAC remained stable over time, indicating that time alone did not alter the tissue's condition. This stability ensures that all subsequent variations in the OAC observed in treated groups can be attributed solely to the effects of the OCAs themselves, rather than to other environmental effects, such as tissue drying.

When comparing OAC values across all formulations that effectively decreased the attenuation regardless of experimental time, we observed that the peak clearing efficiency depends on both the time point and the agent (see Figure 5). Maximum performance reached was 11.8% ± 1.8% for the PEG, 15.7% ± 3.7% for FRU+PROP, 16.8% ± 4.8% for FRU+PEG+PROP, 20.4% ± 3.9% for TA, and 23.8% ± 1.7% for the PEG+OA. This best performance was achieved at 12 min, except for PEG+OA, which achieved it at 15 min. In contrast, the remaining formulations either failed to promote optical clearing or negatively impacted tissue transparency. Specifically, MO showed negligible effects, maintaining OAC values near baseline levels (0.7% ± 2.7%), and OA and the PEG+PROP mixture led to an increase in the OAC, with increments of 8.2% ± 2.7% and 5.2% ± 5.6%, respectively.

Despite the use of MO as index matching to reduce the reflection at the skin surface and improve the light penetration [36, 39], this effect did not translate into lower OAC values. This may be due to the difficulty of the MO to trespass the stratum corneum (SC) barrier. Lipophilic and hydrophobic agents, such as MO, are theoretically capable of clearing the lipid‐rich epidermis. However, they encounter significant physiological barriers [36]. The SC, characterized by its dense intercellular lipid matrix, remains the primary impediment of most topically applied agents [8, 17]. While MO may modulate the refractive index at the tissue surface, it lacks the properties of a chemical penetration enhancer. Consequently, it cannot effectively disrupt the SC barrier to reach the deep dermis without the assistance of physical methods or synergistic chemical formulations [7, 36].

The use of OA, a well‐known permeation enhancer [40], led to an increase in the OAC. Its individual effect was evaluated specifically to determine whether OA alone induces any variation in the attenuation coefficient. Interestingly, the associated use of OA and PEG achieved the best performance among all the tested OCAs, demonstrating the synergistic interaction between the two agents, validating the hypothesis that oleic acid acts as a lipophilic permeation enhancer. Temporarily disrupting the lipid bilayer structure of the stratum corneum, it allows PEG to reach deeper dermal layers than in its pure form. On the other hand, when PEG was formulated with propanediol, which is also a permeation enhancer, the mixture failed to yield any significant clearing benefit. Despite the individual clearing potential of PEG, the presence of PROP appears to antagonize its performance. Interestingly, the inclusion of FRU in the formulations (FRU+PROP and FRU+PEG+PROP) restored its optical clearing efficacy. These multi‐component mixtures promoted a significant reduction in OAC, suggesting that FRU may act synergistically with PROP.

Fructose is a monosaccharide commonly used in skin formulations as a moisturizing and humectant agent due to its ability to attract and retain water, thereby maintaining skin hydration [41]. However, it requires a permeation enhancer to effectively reach deeper tissue layers. In our study, we employed 1,2‐propanediol, an FDA‐approved substance, as a penetration enhancer to facilitate fructose delivery into the skin. This formulation induced a reduction in the OAC immediately upon application, maintaining a near‐constant OAC throughout the remainder of the experiment.

When PEG is added to the fructose and propanediol mixture (FRU+PEG+PROP), a delayed response was observed, with significant clearing becoming effective after 3 min. Despite this delay, at the point of maximum efficacy (12 min), both formulations achieved comparable performance. In contrast, the use of PEG alone did not immediately reduce the OAC. Instead, the reduction occurred gradually, reaching its minimum value after 12 min. This delayed action may be attributed to the molecular size of PEG, which hinders its ability to permeate the SC and reach deep tissue [42]. Another possible explanation for the OAC variation in the absence of a permeation enhancer is the occlusion effect: PEG may induce transepidermal water loss, thereby increasing hydration in deeper tissue layers and leading to a gradual decrease in OAC.

The overall best performance was achieved by the PEG+OA combination. Although PEG alone was somewhat effective, its association with OA significantly enhanced its clearing capacity. In dermatological formulations, OA functions both as a moisturizing agent and a potent permeate enhancer, disrupting the lipid bilayer of SC to facilitate the deep penetration of active ingredients. The addition of OA optimized the PEG dynamics, accelerating the reduction of the OAC. Peak performance for this formulation was reached at 15 min. More importantly, this experimental group exhibited greater uniformity, further highlighting the consistency of the optical clearing effect.

The superior performance of the PEG+OA combination aligns with the findings of Zaytsev et al. (2020) [36], who explicitly state that the PEG/OA (80/20%) mixture yielded the highest values for both optical depth of detection and penetration. The underlying mechanism involves OA‐induced disruption of the lipid organization within the SC. By increasing the fluidity of SC lipid bilayers, OA facilitates the deep diffusion of PEG molecules [8, 36]. Our observed peak performance at 15 min is consistent with studies suggesting that while chemical enhancers accelerate clearing, the combination of PEG+OA with physical assistance (such as sonication) can achieve maximum penetration in as little as 2 min [36]. Furthermore, Varaka et al. reported that combining propylene glycol with OA reduced attenuation by 43%, the highest value among the agents they tested [16].

Regarding the molecular dynamics of PEG, Berezin et al. reported that, due to its chain length, PEG can interact with two molecular “pockets” of collagen simultaneously [43]. While this increases the interaction energy and stabilizing effect, it also results in slower initial diffusion kinetics compared to smaller molecules. Consequently, PEG alone exhibits limited permeation through an intact SC and requires a co‐intensifier to be effective within a short time frame in in vivo or thick‐tissue applications [8, 36]. Without such enhancers, the attenuation coefficient decreases only marginally and superficially [34].

Finally, the occlusive effect observed in our study (characterized by increased hydration in deeper layers due to the reduction of transepidermal water loss) is a recognized mechanism for viscous agents such as PEG and petrolatum. These substances can promote epidermal hydration and even transiently increase epidermal thickness, which contributes to the complex optical changes observed during the clearing process [16].

Modern literature increasingly focuses on multicomponent mixtures to optimize optical clearing. Studies by Shi et al. and Guo et al. successfully used the FPT mixture (FRU, PEG, and thiazone), demonstrating that adding sugars (such as fructose or sucrose) to polyalcohols significantly enhances contrast and penetration depth [9, 13]. These findings support the trend of using complex formulations to overcome the limitations of single‐agent applications.

Although our results indicate that the PROP+PEG combination alone was relatively ineffective, Wen et al. reported that PEG combined with PROP increased tissue reflectance threefold within 15 min [17]. This discrepancy may be attributed to the method of application: while our study focused on passive topical delivery, the study mentioned employed physical assistance, specifically massage, to facilitate agent infiltration [17]. This suggests that for certain viscous formulations, such as PEG, there are transitions from passive diffusion to active transport.

Tartrazine in aqueous solution was recently demonstrated to be an effective OCA by Ou et al. [35]. Its mechanism of action involves the selective absorption of visible light in the blue region, thereby increasing the refractive index of the aqueous solution at longer wavelengths, as described by the Kramers–Kronig relations. This process aligns the refractive index of the interstitial water more closely with that of the tissue's lipids, thereby reducing scattering and promoting effective, reversible optical clearing. In their work, the authors achieved an exceptionally high clearing effect. However, they utilized subcutaneous injection strategies to achieve these results.

OCA injections are not suitable for all clinical scenarios due to their invasive nature, bioavailability issues, and excretion problems. Therefore, we evaluated the performance of TA through topical application. Under these conditions, TA exhibited the second‐best performance among all the tested OCAs (see Figure 5). It showed a gradual reduction in the OAC, reaching its minimum at 12 min. This slow dynamic is likely attributed to the time required for the water solution to permeate the skin barrier. Notably, the efficacy of TA in the water solution was superior to or comparable to that of the formulation containing specific permeator enhancers, highlighting the potential benefit of future studies that combine TA with chemical permeators to further accelerate and improve its clearing effect.

In our study, we observed that MC consistently yielded higher or comparable OAC values than the control group, depending on the magnitude of compression applied. At low pressures (1.6–3.1 kPa), a significant increase in the OAC was observed. This may be attributed to the rapid expulsion of interstitial water, which increases the packing density of collagen fibers and, consequently, elevates the local scattering density.

At intermediate pressures (4.7–23.5 kPa), the OAC remained constant and did not differ statistically from the control group, suggesting that a state of structural equilibrium may be reached. However, the secondary increase in OAC at higher pressures (31.4–47 kPa), although not statistically significant, likely results from a significant reduction in sample thickness and the resulting physical crowding of cellular and fibrous components, which outweigh the clearing effects of water displacement. The underlying mechanism involves structural modification and the redistribution of tissue components. Specifically, compression alters layer thickness and the volumetric fraction of constituents such as water, proteins, and lipids. These changes lead to local reorganization of scattering elements, directly modulating the tissue's optical properties [26].

Our findings partially align with existing literature while highlighting the complexity of the response. Su et al. found that within the 0–20 kPa range, the total attenuation coefficient in the epidermis increases with pressure, coinciding with a reduction in epidermal thickness [27]. They noted that the attenuation coefficient in the upper dermis initially decreases, then increases again in the deeper dermis. Furthermore, Bachour et al. demonstrated that low pressures (4 and 8 kPa) significantly enhance light penetration in vivo, thereby decreasing the attenuation coefficient of the human forearm dermis by at least 1.0 mm^−1^ [5]. Consequently, our findings did not provide a strong justification for a direct head‐to‐head analysis between these MC and chemical OCA clearing methods in this context. Furthermore, no significant linear correlation was found between mechanical compression and the OAC (*r* = 0.11).

Our study focused on comparing the immediate effectiveness of topically applied OCA formulations and mechanical compression (MC). A limitation of this work is that it did not assess the washout effect. Assessing clearing reversal and agent clearance is essential for protocols involving prolonged light‐tissue interactions, particularly in in vivo settings where tissue metabolism and systemic circulation may influence OAC dynamics. Furthermore, the mechanical compression evaluated here was limited to static pressure. Thus, the washout kinetics of OCAs and the impact of dynamic compression regimes warrant investigation in future studies. Moreover, the present study utilized porcine skin from the ventral abdominal region, which typically exhibits a SC thickness of approximately 15 μm. Given that the SC is the primary rate‐limiting barrier to topically applied agents, future research should evaluate how different regional skin thicknesses and structures affect the clearing efficacy of the proposed OCA formulations.

Additionally, in our work, an exponential decay model was employed to derive a single‐metric comparison of the OAC across the different experimental groups. Future research could utilize alternative models to obtain depth‐resolved OAC profiles [44, 45], which would enable an estimation of the maximum depth at which the clearing effect occurs. Exploring these advanced modeling approaches could provide deeper insights into the spatial distribution of tissue transparency.

## 5 Conclusion

Our results demonstrate that, although MC is practical for clinical applications, it does not significantly reduce optical attenuation in the skin. In contrast, topically applied OCA formulations, particularly PEG+OA, TA, and FRU + PEG+PROP, effectively enhanced light penetration, producing a more pronounced and sustained reduction in the OAC. Notably, a neat TA solution achieved a level of efficacy comparable to other chemical OCAs while offering the advantage of an aqueous formulation. These findings indicate that the effectiveness of optical clearing is strongly dependent on the physicochemical properties of the applied agents. While PEG‐based formulations improve optical transparency, they may also induce tissue dehydration and structural alterations due to their high osmolality. In comparison, skin topically applied TA provides optical clearing without these adverse effects, suggesting a more favorable balance between efficacy and tissue preservation. Moreover, its performance could potentially be further improved through the combination of TA with targeted skin permeation enhancers. These results contribute to the development of safer and more effective strategies for enhancing light‐based diagnostic and therapeutic procedures in dermatological applications.

## Funding

This work was supported by Fundação de Amparo à Pesquisa do Estado de São Paulo (2017/21851‐0, 2022/03556‐9), Conselho Nacional de Desenvolvimento Científico e Tecnológico (406761/2022‐1, 314517/2021‐9, 440228/2021‐2, 446212/2024‐5, 444113/2024‐0, 385641/2025‐7), Coordenação de Aperfeiçoamento de Pessoal de Nível Superior (888881083226/2024‐1), and Comissão Nacional de Energia Nuclear (2020.06.IPEN.04).

## Conflicts of Interest

The authors declare no conflicts of interest.

## Data Availability

The data that support the findings of this study are available from the corresponding author upon reasonable request.
